# Cryo-EM structure and functional landscape of an RNA polymerase ribozyme

**DOI:** 10.1073/pnas.2313332121

**Published:** 2024-01-11

**Authors:** Ewan K. S. McRae, Christopher J. K. Wan, Emil L. Kristoffersen, Kalinka Hansen, Edoardo Gianni, Isaac Gallego, Joseph F. Curran, James Attwater, Philipp Holliger, Ebbe S. Andersen

**Affiliations:** ^a^Interdisciplinary Nanoscience Center, Department of Molecular Biology and Genetics, Aarhus University, Aarhus 8000, Denmark; ^b^Division of Protein and Nucleic Acid Chemistry, Medical Research Council, Laboratory of Molecular Biology, Cambridge CB2 0QH, United Kingdom

**Keywords:** RNA, polymerase, ribozyme, cryo-EM, fitness landscape

## Abstract

The emergence of an RNA replicase capable of self-replication is considered an important stage in the origin of life. RNA polymerase ribozymes (PR) - including a variant that uses trinucleotide triphosphates (triplets) as substrates - have been created by in vitro evolution and are the closest functional analogues of the replicase, but the structural basis for their function is poorly understood. Here we use single-particle cryogenic electron microscopy (cryo-EM) and high-throughput mutation analysis to obtain the structure of a triplet polymerase ribozyme (TPR) apoenzyme and map its functional landscape. The cryo-EM structure at 5-Å resolution reveals the TPR as an RNA heterodimer comprising a catalytic subunit and a noncatalytic, auxiliary subunit, resembling the shape of a left hand with thumb and fingers at a 70° angle. The two subunits are connected by two distinct kissing-loop (KL) interactions that are essential for polymerase function. Our combined structural and functional data suggest a model for templated RNA synthesis by the TPR holoenzyme, whereby heterodimer formation and KL interactions preorganize the TPR for optimal primer–template duplex binding, triplet substrate discrimination, and templated RNA synthesis. These results provide a better understanding of TPR structure and function and should aid the engineering of more efficient PRs.

## Significance

The RNA world hypothesis posits that RNA self-replication played a foundational role at the origin of life. To explore this hypothesis, researchers have used selection methods to discover RNA catalysts (ribozymes) with polymerase activity. Some such polymerase ribozymes can replicate parts of their own sequence but little about their three-dimensional structures was known. Here, we describe the determination of the structure of one such polymerase ribozyme using cryo-EM and map its functional landscape. This revealed how this ribozyme functions as an RNA heterodimer and suggests how this structure may aid in copying RNA from RNA templates. Our study provides insight on the potential working mechanism of an RNA replicase and highlights RNA’s functional and structural versatility.

RNA catalysts (ribozymes) occupy central structural and catalytic roles in the function of modern cells including tRNA processing (RNaseP), RNA splicing [spliceosome, group I/II self-splicing introns (SSIs)], and translation (ribosome peptidyl transferase center) (1). In addition, a much wider variety of ribozyme activities not found in nature have been discovered by in vitro evolution, including polymerase ribozymes (PR) that are capable of synthesizing a complementary strand on an RNA template (2–10). Their capacity for RNA-catalyzed RNA-templated synthesis and replication may give rise to a replicase activity enabling RNA self-replication, a process postulated as a central pillar of life’s first genetic system (11, 12).

The earliest examples of nascent PR activity were found in SSI ribozymes, in particular a variant of the *sunY* SSI ribozyme, which allowed single nucleotide triphosphate (NTP) extension (13) or the iterative ligation of RNA oligonucleotides on a complementary strand (14) including assembly of one of its subunits from RNA oligonucleotides (15, 16). The same *sunY* SSI ribozyme was also shown to incorporate short RNA trinucleotide substrates (17) but with relatively low fidelity.

The in vitro evolution of the class I ligase (cIL) ribozyme (18) led to a more fully developed PR activity (19), which after further optimization could incorporate up to 14 NTPs in a template-dependent manner (2). The polymerase activity of this first “true” PR was progressively improved by in vitro evolution to enable the synthesis of long RNAs (100 to 200 nts on some RNA templates) (4, 7) as well as the synthesis of functional RNAs including a hammerhead ribozyme (3), tRNA (5), Broccoli fluorescent aptamer (10) and the progenitor cIL ribozyme itself (8). Recently, a variant utilizing trinucleotide triphosphates (triplets) as substrates [a triplet polymerase ribozyme (TPR)] was discovered (10). This TPR emerged as a heterodimer from in vitro evolution and displayed a remarkable ability to copy structured RNA templates including segments of its own sequence (10) as well as circular RNA templates by rolling circle synthesis (20).

However, our understanding of PR function is encumbered by a lack of structural information beyond the progenitor cIL ribozyme (18, 21, 22). While the cIL crystal structures provided insights into the mechanism of phosphodiester bond formation and cIL interaction with the RNA substrate, it is unclear to what extent these features would be retained in PRs, which diverge from the cIL not only by a number of mutations in the ribozyme core, but also by 5′- and 3′-extension sequences. A better understanding of how PRs perform accurate substrate selection, general RNA template interaction, and templated RNA synthesis, would therefore benefit from the structure of an active PR.

Here, we report the cryogenic electron microscopy (cryo-EM) structure of an active PR, specifically the complete, heterodimeric TPR apoenzyme determined at its optimal functional magnesium ion concentration ([Mg^2+^] = 100 mM), augmented by a comprehensive fitness landscape of TPR function. Our results reveal the molecular anatomy of the two polymerase subunits and the geometry and functional importance of their mutualistic association. Our fitness landscape analysis provides a fine-grained mapping of nucleotides important for polymerase function, which together with the structural data define the functional core domains of the TPR and provide the foundation for a model for the TPR holoenzyme consistent with all functional data.

## Results

### Cryo-EM Structure of Optimized TPR Heterodimer.

**Fig. 1.**
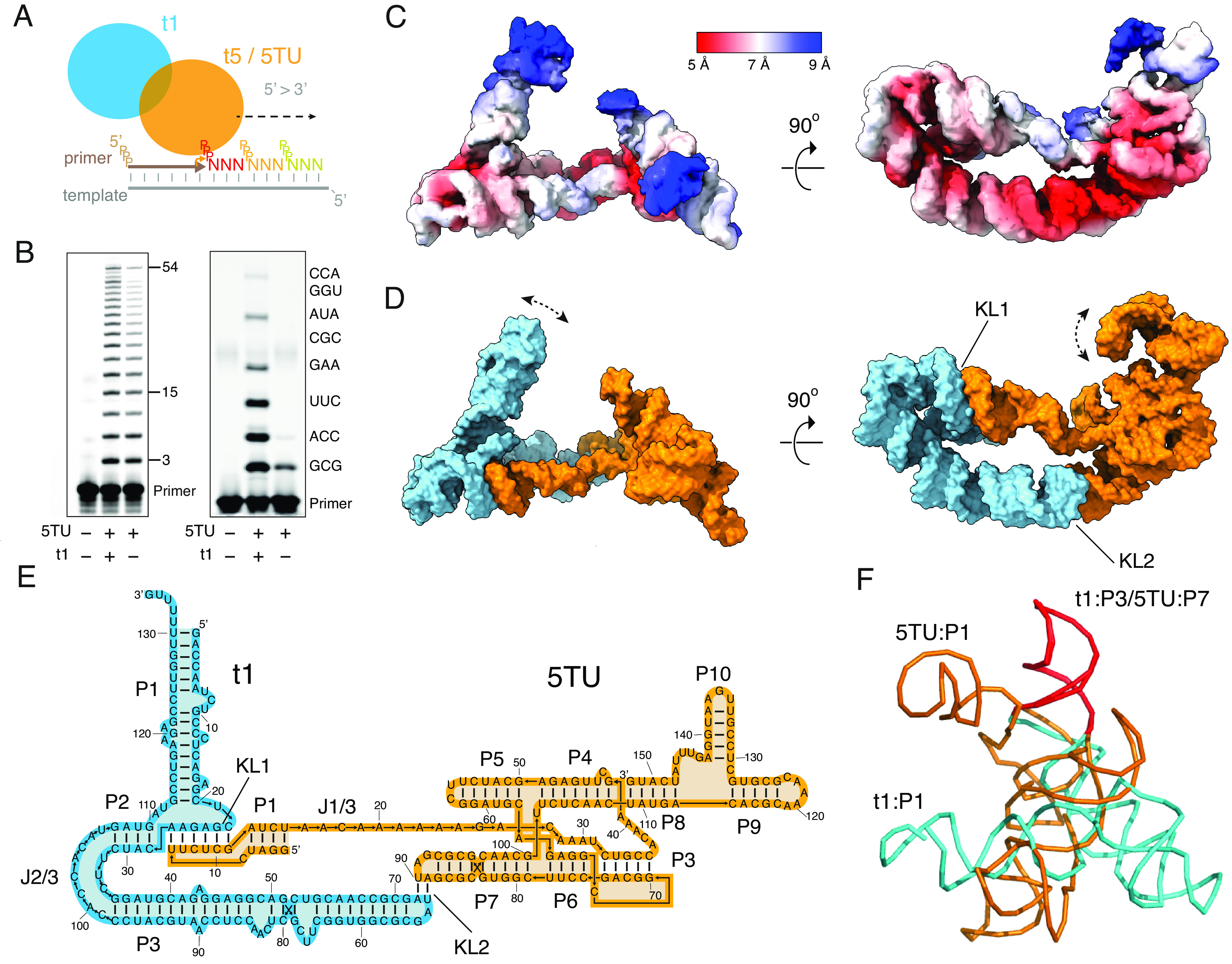
Structure of the TPR. (*A*) Schematics of the TPR heterodimer consisting of the catalytic subunit 5TU and scaffolding subuint t1 acting on primer, template, and triplet substrates. (*B*) Polymerization activity of 5TU alone or in combination with t1 in copying a template encoding (GAA)_18_ after 15 h or eight distinct triplets after 21 h. (*C*) Cryo-EM reconstruction at 5 Å global resolution (EMD-40984) shown in two perpendicular views colored by local resolution estimates. (*D*) Atomic model (PDB: 8T2P) in surface representation shown in two perpendicular views colored by subunit: 5TU (orange), t1 (cyan). Main modes of movement are indicated by double-headed dashed arrows. (*E*) Secondary structure diagram for the TPR heterodimer consisting of subunits 5TU (orange) and t1 (cyan). Helix domains (P), kissing loops (KL), and longer joining regions (J) are annotated. (*F*) Structural alignment of t1 P3 and 5TU P7 stems shows major structural divergence between the two subunits.

We began by seeking to improve activity and stability of the original TPR consisting of a catalytically active subunit (t5) and a catalytically inactive subunit (t1) (Fig. 1*A*) (10). To this end, we first executed further rounds of in vitro evolution using an adaptation of a previously described tethered template selection scheme (10) (*SI Appendix*, Fig. S1*A*). In this experiment, we identified two activity-enhancing mutations in t5 (ΔU38 and C110U) and combined them with three more t5 mutations (U117C, U132C, and U148A) identified in separate selection experiments (to be described elsewhere) (*SI Appendix*, Fig. S1*B*). This t5 (ΔU38, C110U, U117C, U132C, and U148A) variant was named 5TU and exhibited superior triplet polymerase activity (*SI Appendix*, Fig. S1*C*). 5TU showed 1.7-fold enhancement by t1 in copying longer repetitive templates (Fig. 1 *B*, *Left*) and a strong dependence on the t1 accessory subunit on complex templates (Fig. 1 *B*, *Right*).

Next, we sought to obtain structural information on the TPR consisting of 5TU and t1 subunits (5TU+t1 apoenzyme) in its active form at an optimal Mg^2+^ concentration of 100 mM (*Materials and Methods* and *SI Appendix*, Figs. S2–S6 and Table S1) using cryo-EM. Attempts at capturing the TPR in a substrate-bound conformation (holoenzyme) led only to reconstruction of the apo form of the TPR indicating that the template was binding too transiently to allow 3D reconstruction. We obtained a 5.0-Å map of the TPR heterodimer apoenzyme with a local resolution varying from 5 to 9 Å (EMD-40984), indicative of high flexibility of some ribozyme domains (Fig. 1*C*). The latter was verified by 3D variability analysis (3DVA) (*SI Appendix*, Figs. S7 and S8). The 5TU subunit was modeled by aligning the cIL core and fitting the 5′ and 3′ extension domains in the cryo-EM map. The remaining density in the cryo-EM map suggested an alternative fold of the t1 subunit. Reevaluation by secondary structure prediction revealed an extended fold with three main domains (P1-3) (*SI Appendix*, Figs. S9 and S10), that could be unambiguously placed in the cryo-EM map. The joining (J) and loop (L) regions of 5TU and t1 were assembled de novo using DRRAFTER (23) (*SI Appendix*, Fig. S11). The final model of the heterodimer was refined using molecular dynamics and energy minimizations (*Materials and Methods*) and reached a map-to-model cross-correlation of 7.3 Å at FSC = 0.5 and 5.6 Å at FSC = 0.143 (Fig. 1*D* and *SI Appendix*, Fig. S13). The model (PDB code: 8T2P) can be described in a secondary structure diagram that shows helix regions and kissing-loop (KL) interactions (Fig. 1*E*) and a map including tertiary interactions (*SI Appendix*, Fig. S14).

The model revealed the overall structural anatomy of the TPR to resemble an upturned left hand, with the thumb formed by the t1 subunit and fingers formed by the 5TU subunit at an approximate angle of 70°, and the palm formed by a bipartite interaction of the subunits through two distinct kissing loops (KL1, KL2) (Fig. 1*D*). The 5TU subunit comprises the catalytic core domains P3-7, the template binding strand J1/3, and peripheral domains P1+P8-10. In contrast, the noncatalytic accessory subunit t1 adopts an extended secondary structure that contains only three main stem domains P1-3. Thus, the secondary and tertiary structures of the individual 5TU and t1 subunits have diverged radically, which was unanticipated, since both subunits are derived from the same starting sequence by evolution (10) and have only diverged by seven mutations in the core region (*SI Appendix*, Fig. S10*A*). The only common structure left is a 22-bp segment corresponding to the apical hairpins of the 5TU:P7 and t1:P3 domains, which is involved in the symmetrical KL2 interaction (Fig. 1*F* and *SI Appendix*, Fig. S15).

Further analysis of the cryo-EM data showed that local refinement of the two subunits does not lead to improvements in the resolution (*SI Appendix*, Figs. S5 and S6) suggesting that the bipartite KL interaction is rigid or that both subunits have comparable internal flexibility. Two major conformations of the t1:P1 stem were isolated during particle classification (*SI Appendix*, Fig. S8) and 3DVA revealed a directional movement of both t1:P1 and 5TU:P10 domains toward the active site (arrows in Fig. 1*D* and Movie S1). We had further investigated the progenitor t5+1 ribozyme (10) at lower (25 mM) Mg^2+^ concentrations and obtained an independently determined 8 Å resolution map that show the same general shape and conformation (*SI Appendix*, Fig. S16) suggesting that the 5TU+t1 structure had not significantly diverged from t5+1 and that Mg^2+^ concentration did not affect the overall ribozyme shape or quaternary structure.

### Fitness Landscape of TPR Heterodimer.

**Fig. 2.**
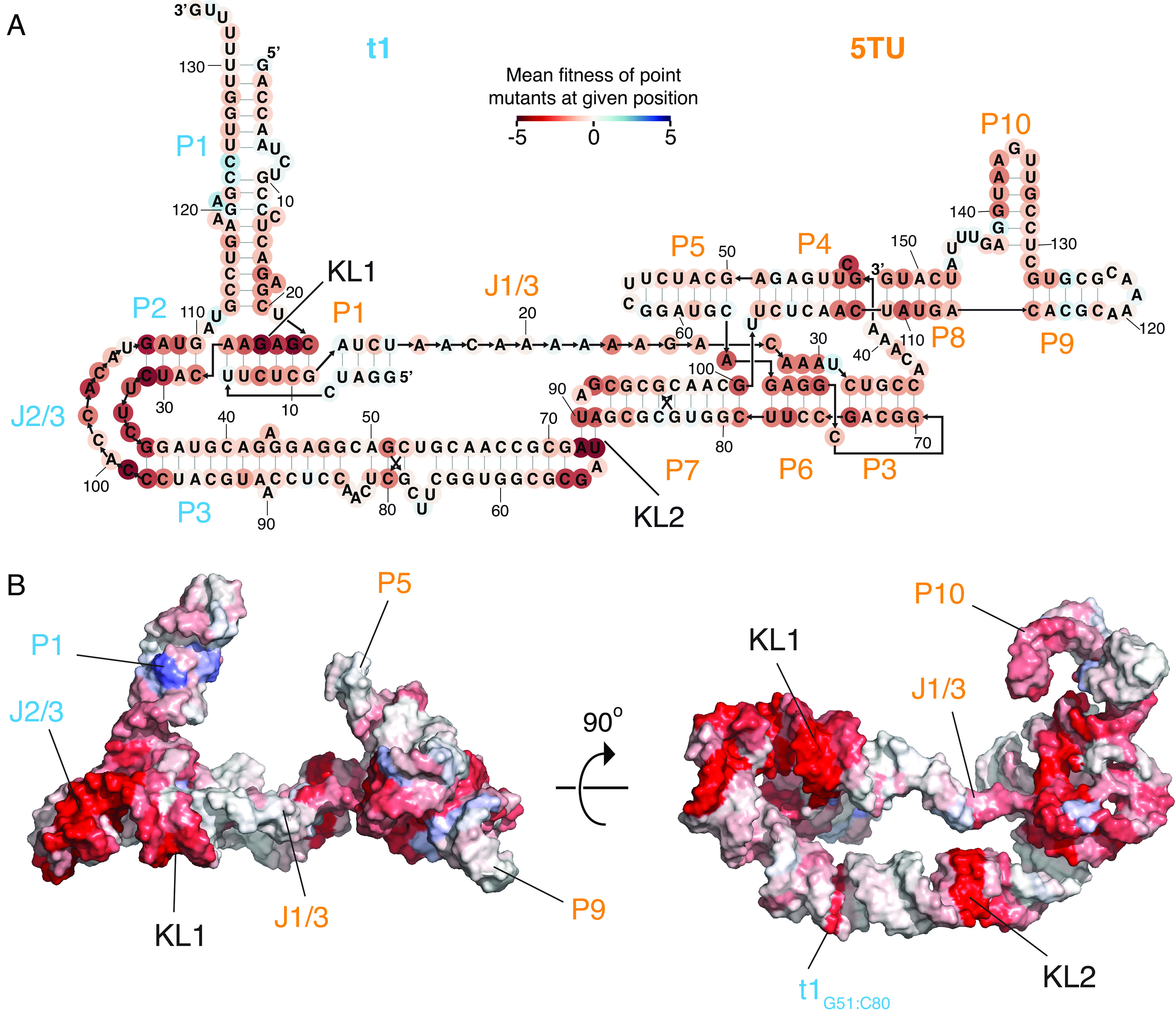
Fitness landscape of the TPR. Average fitness values for a given nucleotide position in TPR secondary structure (*A*) and tertiary structure (*B*) (Movie S2). Mean fitness of point mutations is shown by color scale from red to blue. Names of stems and junctions of t1 and 5TU are colored in cyan and orange, respectively. For full map fitness of individual mutations at all positions, see *SI Appendix*, Fig. S19.

To connect structural features in our 5TU+t1 heterodimer model to TPR function, we performed a comprehensive fitness landscape analysis (24, 25) in triplicate (*Materials and Methods*, Fig. 2, *SI Appendix*, Figs. S17–S22, and Movie S2) by quantification of changes in genotype abundance pre- and postselection for TPR activity; we define ribozyme “fitness” as the log-transformed enrichment of a given genotype relative to the wild-type (wt) 5TU or t1 sequence. After filtering, we obtained relative fitness values for 128,708 ribozyme variants, comprising 79,702 5TU and 49,006 t1 genotypes, providing fitness estimates of all t1- as well as 99.6% of 5TU-single mutants. For both subunits, calculated fitness was strongly correlated across replicates [Pearson coefficient R = 0.89 (5TU)/0.95 (t1), and R = 0.97 (5TU)/0.95 (t1) if only single and double mutants were considered] (*SI Appendix*, Fig. S17*B*).

Next, we analyzed the dataset for global properties and concordance with established TPR function. While mean fitness of both 5TU and t1 mutants was negatively correlated with Hamming distance from wt sequences, the fitness decline was noticeably steeper for 5TU than for t1 (*SI Appendix*, Fig. S18). Furthermore, while the majority of 5TU genotypes showed a much-reduced fitness compared to wt, the t1 fitness distribution–while also negatively skewed–was considerably flatter (*SI Appendix*, Fig. S17*A*). These results are consistent with the highly evolved catalytic 5TU subunit occupying a steeper fitness peak (in a more rugged adaptive landscape) compared to the more recently evolved, noncatalytic t1 accessory subunit.

Fitness landscape analysis further revealed the functional relevance of both known structural features of cIL (21) as well as structural features that are unique to the TPR (Fig. 2 *A* and *B* and *SI Appendix*, Fig. S19). Functionally important structural features known from the cIL structure include the template-binding nucleotides in J1/3 (positions 22 to 24), the active site cytidine in P4 (position 43), and the P6 triple helix-forming adenosines (positions 28 to 30). Novel features of importance to TPR function include the P10 stem (positions 137 to 140), the KL interactions (KL1 and KL2) between the two subunits, as well as the internal loop region of t1 J2/3, J3/2 (positions 99 to 106 and 32 to 34) and a G-C base pair (bp) in t1 P3 (positions 51 and 80). These will be discussed below in relation to the structural analysis.

Analyzing double mutants, we found epistatic interactions in both 5TU and t1 that were negatively biased (*SI Appendix*, Figs. S20 and S21) and rarer in t1 than in 5TU. Moreover, both the proportion of significant epistatic interactions and the magnitude of epistasis, decreased in both subunits (*SI Appendix*, Fig. S22*B*) with increasing physical distance between residues (as predicted from our structural model). Finally, we found that the average epistatic value decreased as the fitness of the first point mutation increased in double mutants of both 5TU and t1 (*SI Appendix*, Fig. S22*A*). All of these trends are consistent with previously determined fitness landscapes of a yeast tRNA (26), and snoRNA (27), suggesting that they may represent general features of RNA structure and evolution.

Although our dataset did not comprehensively capture all double mutants in either ribozyme subunit, many double mutants at predicted base-pairing positions exhibit positive epistasis, (particularly within t1) and point mutations that result in a wobble base pair were consistently higher in fitness compared to base pair-disrupting point mutations (*SI Appendix*, Fig. S23*B*), lending further support to our structural model (*SI Appendix*, Fig. S23 and Tables S2 and S3).

### Structure and Function of Bipartite KL Linkage.

**Fig. 3.**
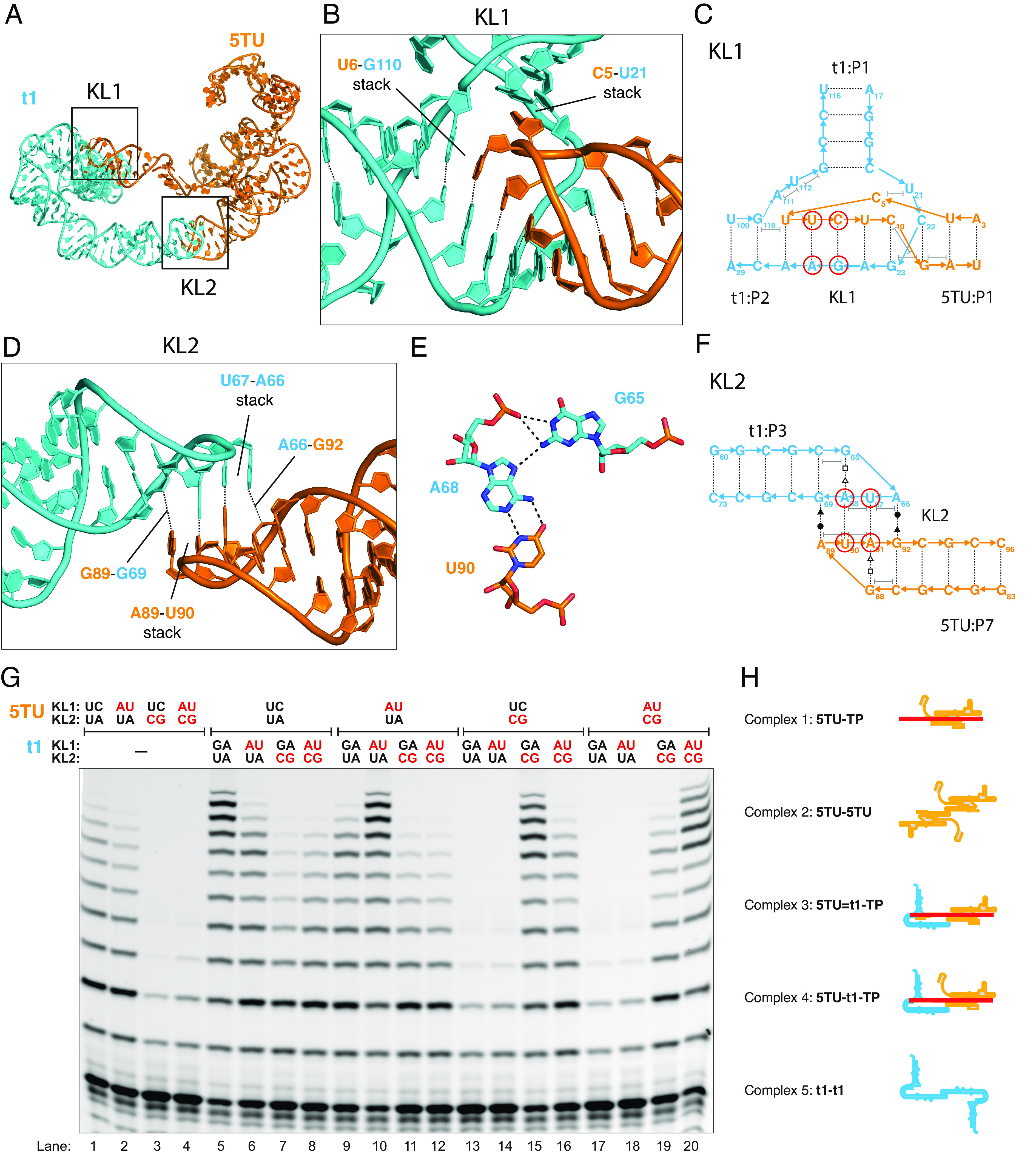
Structure and function of bipartite KL interaction. (*A*) Atomic model of the TPR heterodimer shown in cartoon representation with 5TU in orange and t1 in cyan. KL1 and KL2 are indicated by boxes. (*B*) Zoom on KL1 showing the branched conformation with annotation of the C5:U21 base stack. (*C*) Secondary structure diagram corresponding to panel *B* with annotation of base stacking. Red circles show mutated base pairs used in panel *G*. (*D*) Zoom on KL2. (*E*) Structural detail of KL2 showing U90:A68 base pair and A68:G65 noncanonical interaction. (*F*) Secondary structure diagram corresponding to panel *D* with annotation of non-Watson-Crick base pairs and stacking. Red circles show mutated base pairs used in panel *G*. (*G*) Primer extension activity of TPR with wild type as well as mutant KL sequences visualized by gel electrophoresis. Positions of KL mutations are shown in panels *C* and *F*. Mutation of 5TU:KL1 and in particular KL2 reduce TPR activity, but activity can be restored by compensating mutations in cognate loops in t1. (*H*) List and illustration of the proposed complexes between 5TU, t1, and template-primer (TP).

A striking feature of the TPR structure is that the two divergent subunits are held together by two distinct KL (KL1 and KL2) interactions (Fig. 3*A*, see model in cryo-EM map in *SI Appendix*, Fig. S25). The geometry created by these two KL interactions enforces a rigid, extended conformation of the single-stranded 5TU:J1/3 segment that is clearly visible in reconstructions from all our datasets. Importantly, heterodimer formation is essential for full triplet polymerase activity (Fig. 1*B*) and for primer/template interaction enabling RNA synthesis activity without in-cis tethering of the template, which is needed for most other PR (3).

The structure of KL1 shows a 6-bp interaction between the loop of the 5TU:P1 hairpin and the t1:J1/2 internal loop, which results in a coaxial stack of 5TU:P1, the KL, and the t1:P2 stems (Fig. 3 *B* and *C*). The interaction is reminiscent of a branched KL (28) with several similar features like the bridging over the major groove. The 5TU:C5 that bridges the major groove is observed to base stack with t1:U21 (Fig. 3 *B* and *C*), while the two single-stranded bases A111 and U112 stack underneath the t1:P1 stem. The KL1 base pairing between 5TU:U6-G11 and t1:C22-A27 shows a clear functional signal in the fitness landscape analysis since mutations in these regions are detrimental, and mutations to a GU wobble pair are less severe (*SI Appendix*, Fig. S24*A*). Mutations of the 5TU:C5 to t1:U21 base stack do not show a strong effect on fitness (*SI Appendix*, Fig. S24*A*).

The structure of KL2 is a 2-bp loop-loop interaction between the identical apical loops of 5TU:P7 and t1:P3 (Fig. 3*D*). The 5TU:P7 and t1:P3 have identical terminal GAUA loops as a consequence of the shared evolutionary ancestry between 5TU and t1 (*SI Appendix*, Fig. S10). The KL2 structure shares a high structural similarity to the GACG KL of the Moloney murine leukemia virus (MoMuLV) as determined by NMR (29), which is a palindromic/symmetrical interaction involved in homodimerization of retroviral RNA genomes. However, whereas the 2-bp interaction is formed by two GC basepairs (bps) in the MoMuLV KL, it is formed by two AU bps in KL2. The central AU bps in KL2 are stabilized by hydrogen bonding to the first G of the tetraloop (Fig. 3 *E* and *F*), while the second A of the tetraloop stacks on the KL2 bps and forms an additional interstrand hydrogen bond to the 2’O of the stem GC bp (Fig. 3*F*). Because of the symmetry of the KL2 interaction, the fitness of 5TU and t1 point mutants in the two loops and first bp of the stems are virtually identical, again with mutations to GU wobble pairs being less severe (*SI Appendix*, Fig. S24*B*).

To investigate the role of the KL interactions on TPR activity, we introduced targeted mutation into both KL1 and KL2 and analyzed their functional impact by primer extension assays (Fig. 3*G*). We first examined activity of 5TU in the absence of the t1 subunit. In this scenario, mutation of 5TU:KL1 did only marginally reduce polymerase activity (Fig. 3*G*, compare lanes 1 and 2), indicating that this mutation does not affect the interaction between 5TU and the template–primer (TP) helix (Fig. 3*H*, complex 1). In contrast, mutation of 5TU:KL2 strongly inhibited primer extension (lanes 3 and 4), which we hypothesize is caused by formation of 5TU homodimers that inhibit TP binding (Fig. 3*H*, complex 2). Next, we examined activity of the 5TU+t1 ribozyme. Here, as described earlier (Fig. 1*B*), 5TU gains a boost of primer extension activity (lane 5), and this is retained in the presence of mutually compensatory mutations, which support interaction via KL1, KL2, or both (lanes 10, 15, and 20), since they all support heterodimer formation (Fig. 3*H*, complex 3). When only the KL1 interaction is disrupted (by an AU double mutation) in either 5TU or t1, while the KL2 interaction is maintained, we see a slightly reduced activity (lanes 6, 9, 16, and 19), which can be explained since KL2 is still able to form the heterodimer (Fig. 3*H*, complex 4). When t1:KL2 is disrupted (by a CG double mutation), we see a reduction of activity to that of 5TU alone (lanes 7, 8, 11, and 12), which might be explained by t1 forming strong homodimers and does thus not contribute to the activity boost (Fig. 3*H*, complex 5). When 5TU:KL2 is disrupted (by a CG double mutation) with no compensatory mutation in t1, we see strong inhibition of activity (lanes 13, 14, 17, and 18), which may again be explained by strong 5TU homodimer formation (Fig. 3*H*, complex 2). Taken together, the mutational analysis confirms the importance of the cognate KL1/2 interactions in promoting formation of the fully active TPR heterodimer and highlights the importance of the bivalent KL interaction in favoring the correct heterodimer over possible poorly active homodimers that can be formed through the symmetric KL2 interaction.

### Structure of Catalytic Subunit 5TU.

**Fig. 4.**
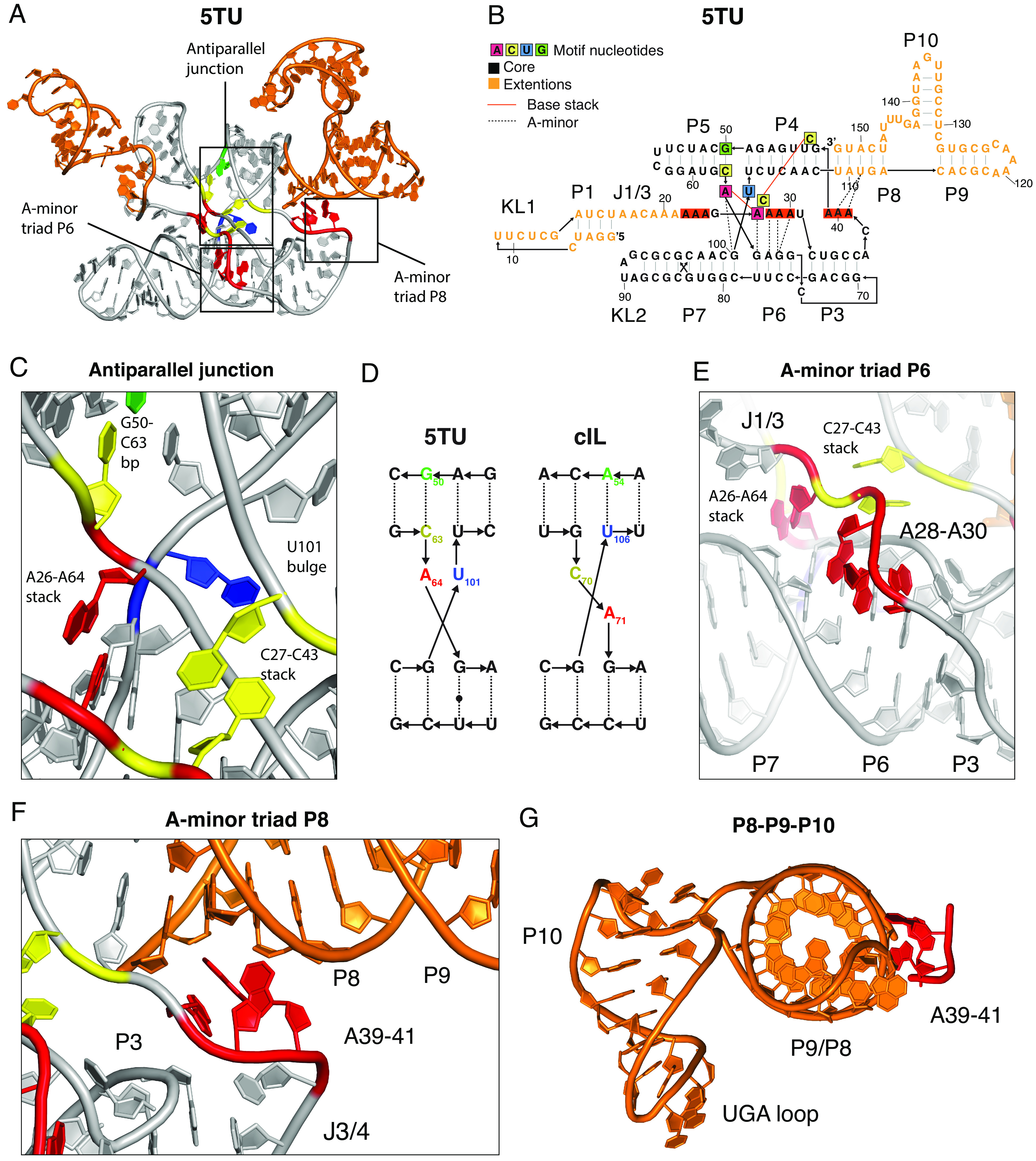
Structural features of catalytic subunit 5TU. (*A*) Atomic model of the 5TU subunit shown in cartoon representation with nucleotides in core motifs and extension regions colored as in panel *B*. Core motifs are indicated by boxes. (*B*) Secondary structure diagram of 5TU with annotation of tertiary interactions and core motif nucleotides. (*C*) Zoom on antiparallel junction and its interaction with J1/3 showing central base pairs, stacks, and bulges. (*D*) Diagram showing the changed junction between 5TU and its progenitor cIL. (*E*) Zoom on A-minor triad interacting with the minor groove of P6. Also shown is the A26-A64 stack that interacts with the minor groove of P6 and P7. Depth fog is used to highlight the motif. (*F*) Zoom on A-minor triad of J3/4 interacting with the minor groove of P8. (*G*) The P8-P10 domain is shown along the helical axis of P8/P9 to show the perpendicular orientation of the P10 helix exposing a 3-nucleotide UGA loop. A39-41 is shown to highlight stabilization from core domain.

The 5TU subunit is a descendant from the cIL with functionally important sequence extensions at both the 5′ and 3′ ends acquired over multiple in vitro evolution experiments to acquire and optimize (triplet) polymerase ribozyme activity. The cryo-EM structure of the 5TU subunit reveals the conformations of the core domain (P3-7) and the 5′ and 3′ extension domains (P1+P8-10) (Fig. 4 *A* and *B*, see model in cryo-EM map in *SI Appendix*, Fig. S26). The 5TU core domain is found to have a similar overall conformation to cIL despite several mutations and the lack of the substrate helices (*SI Appendix*, Fig. S28). Detailed comparison with the crystal structure of cIL shows that there are both similarities and differences in the core structural motifs defining the active site configuration (21) (*SI Appendix*, Fig. S29). A notable difference between cIL and 5TU is found at the antiparallel junction connecting P4-P5 and P6-P7 helices, where the junction has shifted in 5TU due to an A50G mutation (Fig. 4 *C* and *D*). The cryo-EM map supports the formation of a G50-C63 bp and a resulting U101 bulge and this is furthermore supported by the fitness landscape analysis, which shows epistasis for the G50-C63 bp when including GU wobble pairs (*SI Appendix*, Fig. S23*B*). The shifted junction still allows J1/3 to form the important A26-A64 and C27-C43 basestacks of the active site (Fig. 4*C*). A26 of J1/3 forms a base stack with A64 of the P5-P6 junction, which is well supported by both the cryo-EM map (*SI Appendix*, Fig. S26) and fitness data (*SI Appendix*, Fig. S19). C27 of J1/3 forms a base stack with the catalytic C43 bulge of the P4 stem, which is again well supported by the cryo-EM map (*SI Appendix*, Fig. S26) and fitness data showing that while C27 can be mutated (to U), C43 is absolutely conserved (*SI Appendix*, Fig. S19).

A striking feature in our data is the extension of J1/3 into a rigid, single-stranded RNA segment, which is well resolved in the cryo-EM map. The 5′-end of the J1/3 single strand adjoins the P1 hairpin forming the KL1 interaction with t1, while the J1/3 3′-end adjoins the antiparallel junction by the aforementioned base stacks and an A-minor interaction with P6. A28-A30 are observed to form A-minor interactions with P6 similar to the A-minor triad observed in cIL (Fig. 4*E*). This A-triad is highly conserved between 5TU and cIL (*SI Appendix*, Fig. S19) as is the base pairing of the P6 stem (*SI Appendix*, Fig. S23*B*), while a second, novel A-minor triad is observed between A39-A41 in J3/4 and the minor groove of the P8 stem. A39 interacting with the minor groove is supported by mutational data (*SI Appendix*, Fig. S19). In cIL, this interaction cannot happen and is instead formed by a GU base stack, which is not present in 5TU (21) (*SI Appendix*, Fig. S29). The second A-minor triad that in cIL interacts with the substrate helix is placed the same spatial position in 5TU suggesting that the primer–template duplex will likely occupy a similar position.

The 3′-extension domain forms the P8 stem, which branches into the P9 and P10 hairpins. P8 forms a coaxial stack with P4 on one side and P9 on the other. P8:109-113 and P8:148-152 sequence segments are both highly sensitive to mutations (*SI Appendix*, Fig. S19) and supported by epistasis when including GU wobble pairs (*SI Appendix*, Fig. S23*B*). In contrast, the P9 stem is much less sensitive to mutations suggesting a less important role for TPR function. The loop at the end of P9 is modeled as a A119-C123 trans sugar-Hogsteen bp with an A-stack at the 3′ side of the loop. The P10 stem projects from the P8-P9 stem with a four-nt single strand on one side and a direct connection on the other side, which results in a perpendicular orientation of the P10 hairpin (Fig. 4*G*). The hairpin is 6 bp long and ends in a 3-nt loop (positions 135-137) that is modeled as a stack. However, the resolution in this domain is low due to its flexibility and the modeling is only suggestive. The region around the loop of P10 and the junction around P10 is highly sensitive to mutations (*SI Appendix*, Fig. S19) suggesting that P10 is involved in an important functional role. Previously P8-10 had been shown to improve TPR fidelity through minor groove interactions with the triplet substrate (10).

### Structure of Scaffolding Subunit t1.

**Fig. 5.**
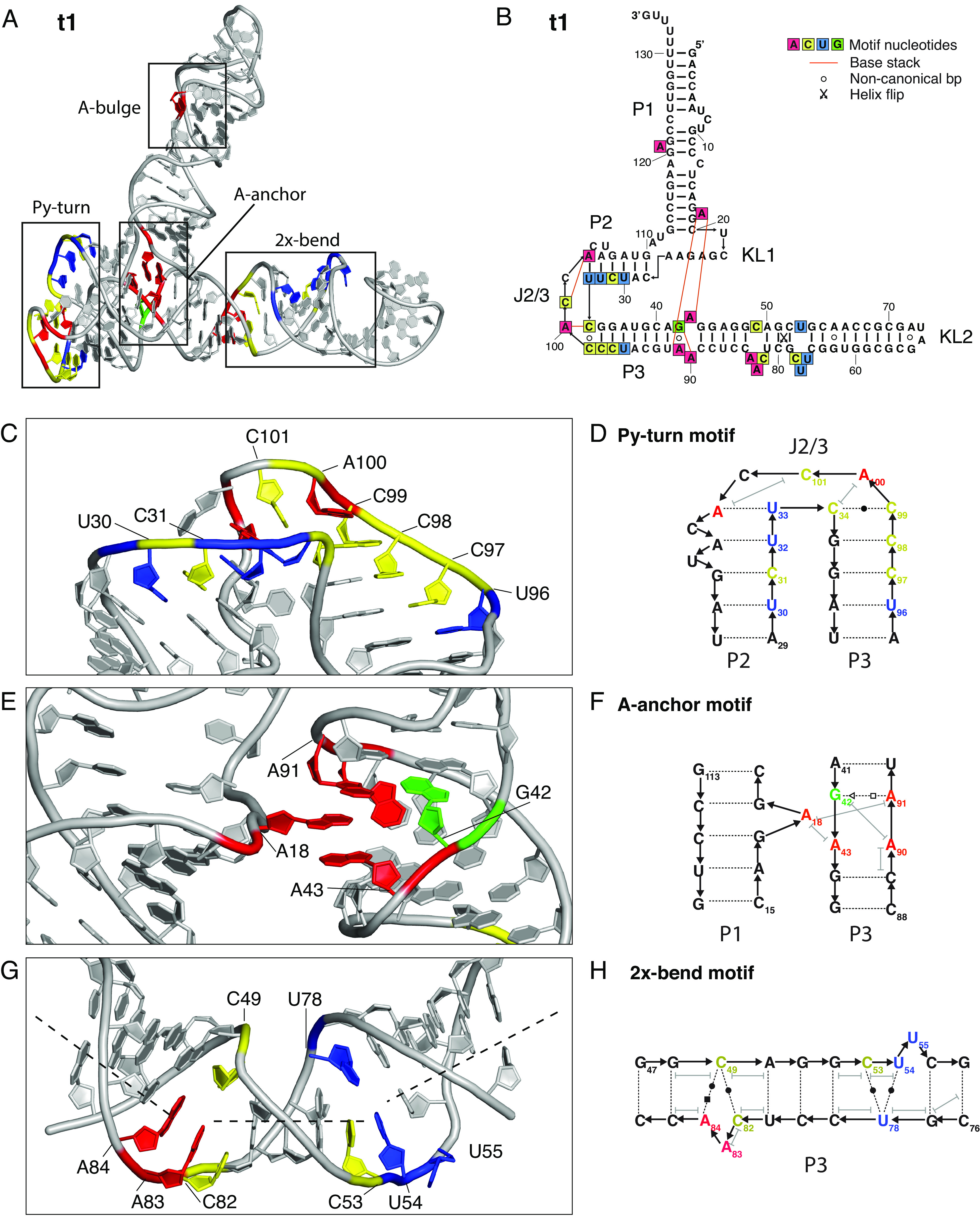
Structural features of scaffolding subunit t1. (*A*) Atomic model of the t1 subunit shown in cartoon representation with nucleotides of core motifs colored as in panel *B*. Core motifs are indicated by boxes. (*B*) Secondary structure diagram of t1 with annotation of tertiary interactions and core motif nucleotides. (*C*) Zoom on Py-turn motif showing Py stretches, noncanonical base pairs, and stacking interactions. (*D*) Diagram of Py-turn motif with annotation of the noncanonical base pair C34-C99 and stacking interactions. (*E*) Zoom on A-anchor motif showing stacking of bulged A18 from P1 in the minor groove of P3. (*F*) Diagram of A-anchor motif with annotation of the noncanonical base pair G42-A91 and stacking interactions. (*G*) Zoom on 2x-bend motif showing asymmetric internal loops with bases colored as in panel *B*. The bending is highlighted by annotation of helical axes by dashed lines. (*H*) Diagram of 2x-bend motif with annotation of the noncanonical base pairs and stacking interactions.

The t1 subunit evolved as a mutualistic parasite to the catalytic domain in the original TPR selection (10) and differs from 5TU through only seven mutations in the core domain and a distinct 3′ extension sequence. Its cryo-EM structure reveals that these changes have caused a major remodeling of both the secondary and tertiary structure. The core domain of t1 now forms an extended structure forming an L-shape (Fig. 5 *A* and *B*, see model in cryo-EM map in *SI Appendix*, Fig. S27) supported by positive epistasis values in our fitness landscape analysis (*SI Appendix*, Fig. S30 and Table S3). The overall t1 structure is composed of three main helix domains: P1 formed by the 5′ and 3′ regions, P2 that coaxially stacks on KL1, and P3 that exposes KL2 at its apical loop. The three helices form a stable connection through two novel structural motifs at an approximate distance of one helix turn. These will be discussed below in detail.

The first connection is formed by a sharp turn of the P2 and P3 helices at the J2/3 and J3/2 internal loop, which we name the “Py-turn” motif (Fig. 5 *C* and *D*), since it is facilitated by two pyrimidine tracts (positions 30 to 34 and 96 to 99) that bend the strands toward each other. The strands are further brought together by the presence of a noncanonical pyrimidine-pyrimidine bp C34-C99. The motif is capped by two bases of the J2/3 region that stacks on the P2 and P3 helices, respectively. Three bases (C102, C104, and U106) remain unpaired with distinct density in the major groove of P2 and at the minor grooves of P2 and P3. The presence of this structural motif is further supported by the fitness landscape analysis showing strong conservation of the pyrimidine tracts as well as the noncanonical C34-C99 bp, C102, and C104 (*SI Appendix*, Fig. S19*A*).

The second connection is formed by a bulged-out A from P1 that inserts into an internal loop of P3, which we name the “A-anchor” motif (Fig. 5 *E* and *F*). The motif is formed at the base of the P1 helix and supports a bridge between the P2 and P3 helices that together with the Py-turn motif orients them in a parallel orientation. The A18 bulge is formed between two C-G bp of P1. A18 stacks between A43 and A91 in the minor groove, which is resolved clearly in the EM map (*SI Appendix*, Fig. S27). The alternative position of A91 is facilitated by a trans-Hogsteen-sugar bp between G42-A91. Density in the major groove further suggests that A90 stacks on G42 in the major groove. The A-anchor motif is furthermore supported by the fitness landscape analysis, which indicates a positive epistatic interaction between A18 and A43 and for base pairs around the symmetric bulge (*SI Appendix*, Fig. S30) and shows that mutations to either nucleotide causes a reduction in fitness (*SI Appendix*, Fig. S19*A*).

The P3 stem has two other bulge regions that cause the stem to bend approximately 120 degrees toward KL2, which we name the “2x-bend” motif (Fig. 5 *G* and *H*). The first bend is formed by sequence segment C82-A84 across from C49 and the second bend is formed by segment C53-U55 across from U78. Together these bend the P3 across the major groove in-between the motifs. Mutations to these bulge regions do not affect fitness (*SI Appendix*, Fig. S19*A*), suggesting that the precise base composition is not important, whereas the position of 3 bases across from one may suffice for the bending. Interestingly, a G51-C80 bp in the stem between the two bulges is heavily affected by mutation and can be partly rescued by mutation to a GU wobble pair (*SI Appendix*, Fig. S19*A*). In P3, there is a noncanonical G58-A75 bp, which is also preserved in the 5TU:P7 as G81-A98. However, the fitness data suggest that this G:A bp is not critical for function (*SI Appendix*, Fig. S19 *A* and *B*).

The P1 stem is well resolved in the cryo-EM map near KL1 and the A-anchor motif, but less well resolved in the top part. Density is observed at A121, which seems to bulge out of the helix between two GC bps in a similar fashion to the A-anchor motif. However, the functional significance of this structure is unclear as mutations in this region seem to have both positive and negative effects on fitness (*SI Appendix*, Fig. S19*A*). Double mutant epistasis in general support the formation of the P1 stem with positive epistasis for positions 13 and 119 indicating a base pair, while positions 8 and 124 show negative epistasis supporting the bulge (*SI Appendix*, Fig. S30). The t1:P1 helix appears to be supported at its base by two key tertiary interactions (the KL1 and A-anchor) that form a hinge allowing the large dynamic movement of t1:P1 (Movie S1). Because of the orientation of the hinge, the movement of the t1:P1 is toward the 5TU active site with potential functional implications discussed below.

### Model of Holoenzyme.

**Fig. 6.**
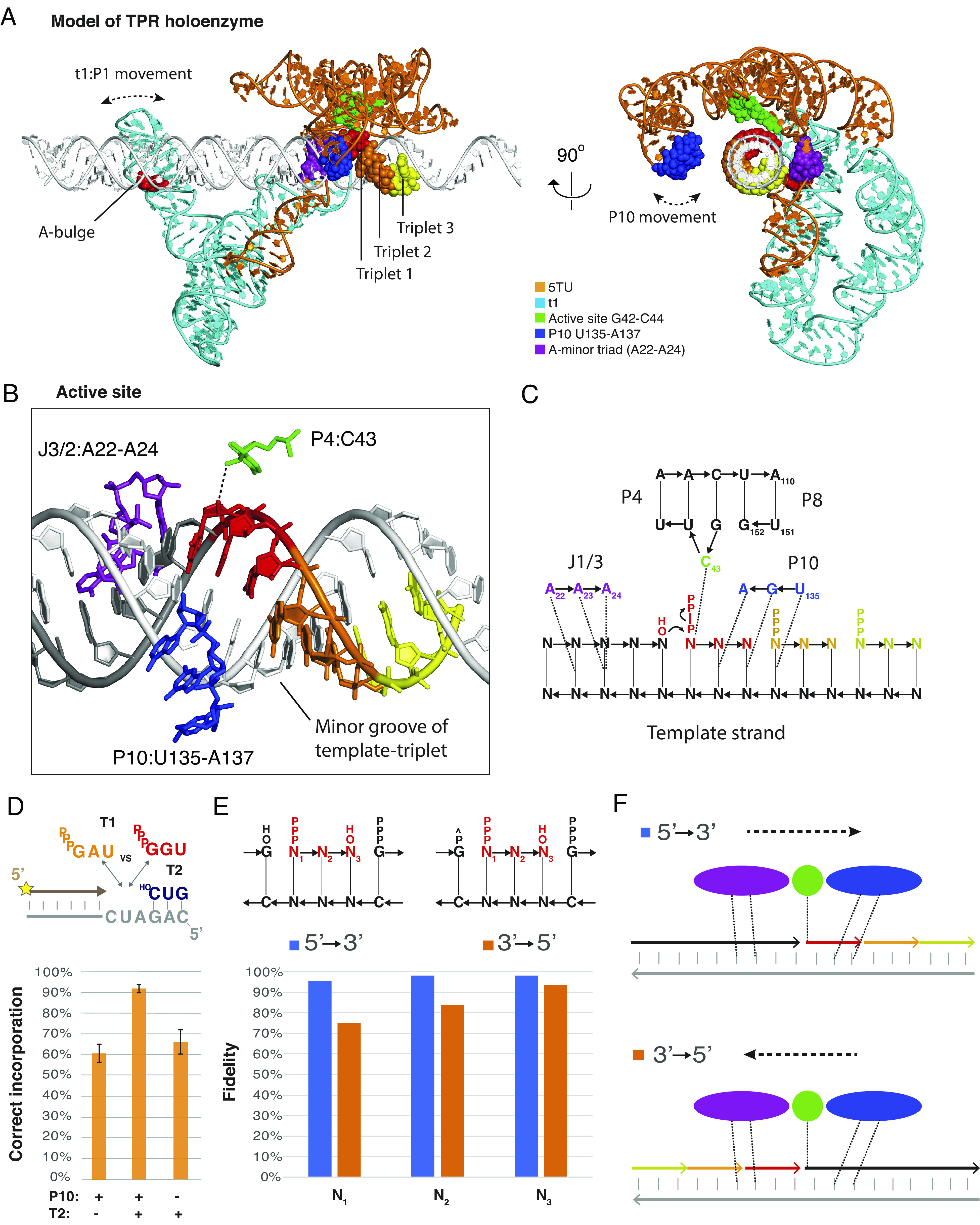
Structural model of the TPR holoenzyme and role of P10. (*A*) TPR model [5TU (orange), t1 (cyan)] with an idealized double-stranded RNA template (white) shown in two perpendicular orthoscopic views. The model was constructed by aligning the cIL structure to 5TU followed by aligning the template to the cIL substrate helix. Core elements are highlighted as spheres: P10 (blue), J1/3 (purple), active site (green), and triplets (red, orange, and yellow). t1:P1 and 5TU:P10 movements from 3DVA are shown as dashed double-headed arrows. (*B*) View of the RNA template in the active site that shows putative minor groove interactions with the primer–template helix and the template–triplet helix. Putative contact sites are shown: active site C43 (green), J1/3 A-minor triad (purple), and P10 loop nucleotides (blue). (*C*) Secondary structure diagram corresponding to panel *B* with putative hydrogen bonds derived from cIL (A-minor triad and C43) and earlier studies (P10). (*D*) TPR fidelity as fraction of correct to incorrect substrate incorporation. Mean ratios are shown, with SDs. For T2−, n = 3. For T2+, n = 2. (*E*) Fidelity of positions 1 to 3 of forward (blue) and reverse (orange) triplet incorporations, as determined by FidelitySeq. ^P indicates a 2′,3′-cyclic phosphate. (*F*) Schematic of 5′ to 3′ forward and 3′ to 5′ reverse polymerization. Colors of interaction domains as in panel B. +1 and −1 triplets are shown in red, +2 in yellow, and +3 in pale green.

Cryo-EM reconstruction of the TPR with template was not possible—likely due to the transient nature of primer–template binding by the TPR. We therefore sought to build a model of the holoenzyme to explore the functional implications of the TPR structure. To this end, we first aligned the catalytic 5TU subunit to the cIL crystal structure (21) and then aligned an extended template–primer helix to the substrate helix in the cIL structure (*SI Appendix*, Figs. S31 and S32). A critical assumption for model building has been that the entry angle of the primer–template duplex in the TPR closely matches that of the substrate helix in cIL. This simple static model (Fig. 6*A*) positions the primer–template duplex, the triplet substrate 5′-triphosphate group, and incoming triplet substrate minor groove in close proximity to likely interacting features of the 5TU subunit (J1/3 segment, active site, and P10 domain, respectively). Additionally, it situates the elongated nascent strand/template duplex near the t1:P1 helix for possible minor groove interaction by an A-bulge of t1:P1 at about 2.5 helical turns (~30 bp) from the active site of the 5TU subunit. 3DVA further suggests movements of the t1:P1 and 5TU:P10 helices toward the active site (Fig. 6*A*, dashed arrows). We hypothesize that the t1:P1 movement may result in a processivity effect by binding to the extended primer–template duplex to promote its progress along its helical axis. The 5TU:P10 movement suggests that it could contact the minor groove of the incoming triplet-template duplex to exert its previously reported fidelity-enhancing activity (10).

In this holoenzyme model, the active site is in close proximity to the J1/3:A22-A24 and the minor groove of the primer–template helix. Furthermore, the model positions the catalytic C43 close to the 5′-triphosphate moiety of the triplet, and the P10:U135-A137 loop close to the minor groove of the triplet-template duplex (Fig. 6 *B* and *C*). In this context, J1/3 is of particular interest because the equivalent positions to 5TU:A22-A24 have been implicated in A-minor interactions with the substrate helix in the cIL structure (21). Indeed, functional data suggest that an extended A-minor triad conformation is essential for full TPR function, with even 2-nt insertion or deletions in J1/3 reducing TPR activity to baseline (*SI Appendix*, Fig. S33). Thus, the t1 domain and its KL interactions may serve to jointly stabilize J1/3 in an outstretched, single-stranded conformation. This may enhance template–primer duplex interactions by reducing J1/3 conformational freedom and secondary structure formation, reducing the entropic cost of template interaction compared to an untethered strand. Analysis of the evolution of the related 52-2 polymerase ribozyme (which uses NTPs as substrates) (6) suggested the emergence of a pseudoknot structure involving P7 and the J1/3 equivalent, which might enhance PR activity via a similar restriction of the conformational freedom of this crucial sequence segment.

A notable feature of the TPR observed previously is its templated RNA synthesis fidelity of 97.4% (per nucleotide position) (10). A significant contribution was ascribed to the P10 [formerly epsilon (10)] domain that—by H-bonding with the minor groove of the 3′ base of the incoming triplet—appears to enhance fidelity compared to 94.5% of a TPR variant that lacks P10 (10). We performed further functional analysis, which suggested that P10 may make even more extensive interactions (Fig. 6*D*): In a challenging pairwise assay the TPR incorporated the correct triplet only 60% of the time when only a single triplet was bound to the template 3′ of the ligation junction. This increased to 92% the presence of a second downstream triplet, but decreased again when using a TPR variant that lacks P10, which suggests possible P10 interactions not just with the incoming triplet in the active site but also with a second downstream triplet. Using substrates of increasing length, P10-dependent fidelity gains are almost entirely restored using a quadruplet (pppN_4_) substrate, with minimal further fidelity gains with longer (pppN_5_ and pppN_6_) substrates (*SI Appendix*, Fig. S34). This suggests that P10 forms functionally important contacts with the substrate-template duplex extending over at least 4 nts. Indeed, our structural model positions P10 and specifically U135, G136 & A137 in close proximity, poised for interaction with the minor groove of the incoming triplet substrate (Fig. 6*B*).

Another remarkable feature of the TPR is its capacity to support noncanonical RNA synthesis modes such as triplet polymerization in the reverse 3′ to 5′ direction (10). To determine the fidelity of the 3′ to 5′ mode of templated RNA synthesis by the TPR we developed an assay, where only the 3′ end of a randomized triplet could be ligated and analyzed it using deep sequencing (FidelitySeq, *SI Appendix*, Fig. S35). The 3′ to 5′ fidelity was reduced to 83.8%, even below the baseline fidelity (ca. 92 to 94%) of 5′ to 3′ synthesis in the absence of the P10 domain (*SI Appendix*, Fig. S36). Although the measured 3′ to 5′ error rate may be inflated due to poor incorporation of AU-rich triplets (*SI Appendix*, Fig. S37), it is clear that 3′ to 5′ fidelity is significantly reduced compared to the canonical 5′ to 3′ synthesis mode. Analyzing the fidelity at individual positions of the triplet, we found that the 3′ to 5′ fidelity was lowest at the N_1_ triplet position (5′-pppN_1_N_2_N_3_) and increased toward the N_3_ position (Fig. 6*E*). These observations can now be rationalized in the light of our holoenzyme model. The model shows that in the 3′ to 5′ mode (with the triplet 5′-triphosphate moiety positioned in the active site) P10 can neither interact with (nor stabilize) the substrate triplet. Instead is positioned to interact with the upstream (3′) primer with no impact on triplet incorporation (Fig. 6*F* and *SI Appendix*, Fig. S38). The increase in fidelity toward the third position may be explained by precise geometrical requirements of positioning of the 3′-OH in the active site.

### Evolution of a Polymerase Ribozyme Heterodimer.

The structure of the TPR comprising a catalytic 5TU and a noncatalytic t1 subunit has some interesting analogies with proteinaceous polymerases such as the HIV reverse transcriptase (RT) holoenzyme heterodimer formed by a catalytic p65 and a noncatalytic p55 (derived from p65). In the HIV RT heterodimer, p55 supports an extended conformation of p65 that allows positioning the primer/template duplex for optimal processive synthesis (*SI Appendix*, Fig. S39). It is tempting to speculate that the noncatalytic t1 RNA subunit may serve a similar function. Indeed, our structure suggests that t1 helps position J1/3 for optimal interaction with the template. Furthermore, our TPR holoenzyme model suggests that RNA templates of 30 nts (or longer) might be able to productively interact with the t1:P1 stem.

The structure of t1 and its bipartite KL interaction with the 5TU subunit offers a potential explanation for the emergence of the mutualistic interaction between the catalytic and accessory subunits during in vitro evolution (10): In the progenitor t1 RNA, the 3′ sequence extension triggered a wide-ranging reorganization of the tertiary fold, abolishing its catalytic activity. Serendipitously, this enabled a kissing loop interaction, which positioned the t1 5′-selection cassette (i.e., template–hairpin, see *SI Appendix*, Fig. S1) near to the active site of a catalytically active RNA (5TU progenitor), allowing for mutualistic exploitation of its activity by t1. Over the course of the selection experiment, t1 gained further mutations to better associate and coevolve with catalytically active subunits, and, in turn, active 5TU progenitor subunits that could exploit t1 complex formation thrived (10). From the KL mutational study, we further find that the bipartite KL interactions likely not only serve to facilitate heterodimerization but also to inhibit nonproductive homodimerization. Thus, mutualism and eventual molecular symbiosis between the two subunits likely emerged by co-opting a parasitic t1 progenitor RNA.

## Conclusion

Our results describe a first structure and comprehensive structure-function analysis of a polymerase ribozyme, providing a framework for a better molecular understanding of templated RNA-catalyzed RNA synthesis, an enzymatic activity widely considered to be fundamental for the emergence of life’s first genetic system. The cryo-EM structural analysis revealed several novel RNA motifs (such as the Py-turn and A-anchor motifs) and RNA motifs with close resemblance to both engineered and retroviral kissing loop motifs. Our results highlight the intricate structural motifs that can be identified through in vitro evolution of large and complex RNA molecules and these may serve as inspiration for rational RNA nanotechnology designs like the RNA origami architecture (30).

## Materials and Methods

Detailed description of the materials and methods used in the study can be found in *SI Appendix*: RNA preparation, cryo-EM data acquisition, single particle image processing and 3D reconstruction, model building, 3DVA, selection library synthesis, in vitro evolution cycle, TPR activity assays, determination of 5TU+t1 adaptive landscape, calculating fitness associated with each genotype, fidelity assay for substrate lengths, FidelitySeq assay, and oligonucleotide syntheses.

## Supplementary Material

Appendix 01 (PDF)

Movie S1. **3D variability analysis of the TPR.** Movie showing 3D variability analysis of the TPR from the 126,690-particle stack shown in Fig. S2.

Movie S2. **Average fitness values on the TPR tertiary structure.** Movie showing the average fitness values for a given nucleotide position on the TPR tertiary structure.

## Data Availability

Cryo-EM map was deposited in the Electron Microscopy Data Bank under accession number EMD-40984 (31). Atomic coordinates have been deposited into the PDB under accession number 8T2P (32). All other data are included in the manuscript and/or supporting information.
